# Supplementary material for: Enhancing Clinical Applications by Evaluation of Sensitivity and Specificity in Whole Exome Sequencing
Source: Int J Mol Sci. 2024 Dec 10;25(24):13250. doi: 10.3390/ijms252413250 (PMC11678496; doi:10.3390/ijms252413250)
Supplement: Supplementary file 1 [file ijms-25-13250-s001.zip › ijms-3316728-supplementary.pdf]

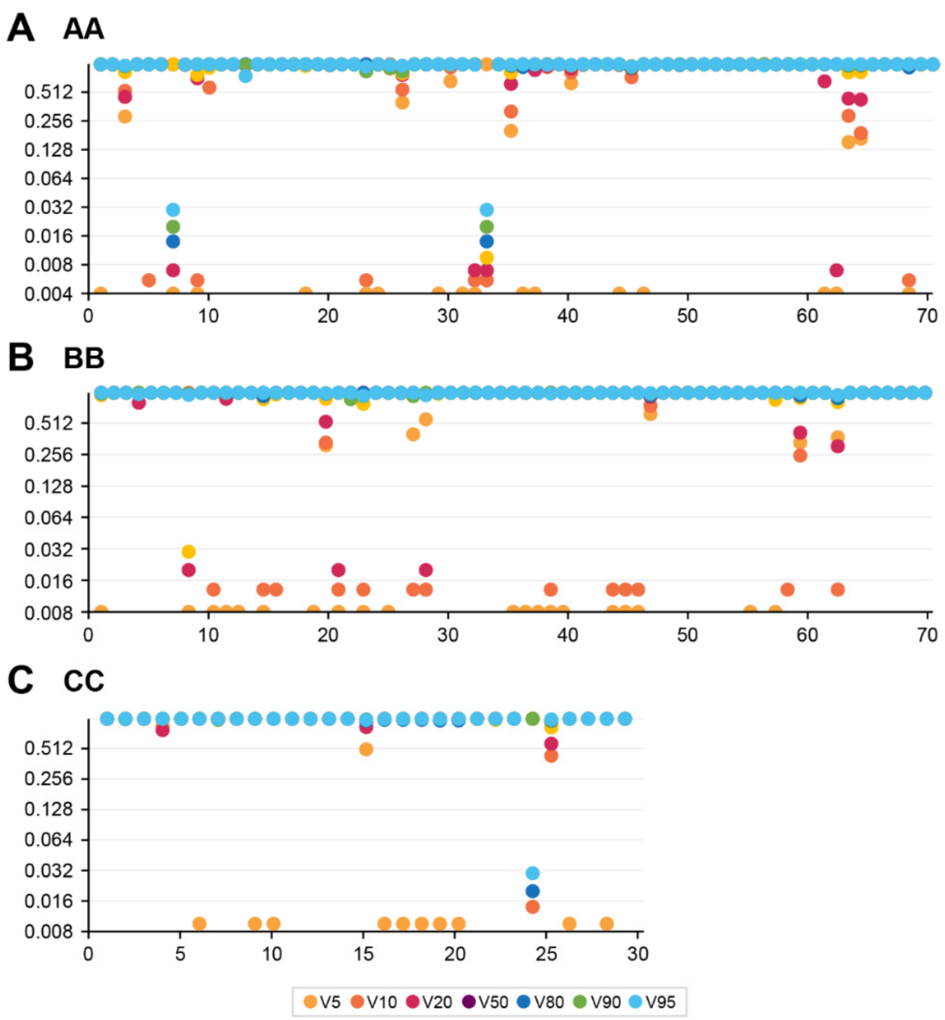

Figure S1. Eliminated N-H variants by trimming. The N-H variants removed by applying VAF cutoffs are illustrated for variants ranging from V5 to V95. A. Eliminated variants in the results from company AA. B. Eliminated variants in the results from company BB. C. Eliminated variants in the results from company CC. The X-axis represents chromosomal position, while the Y-axis shows observed VAF.

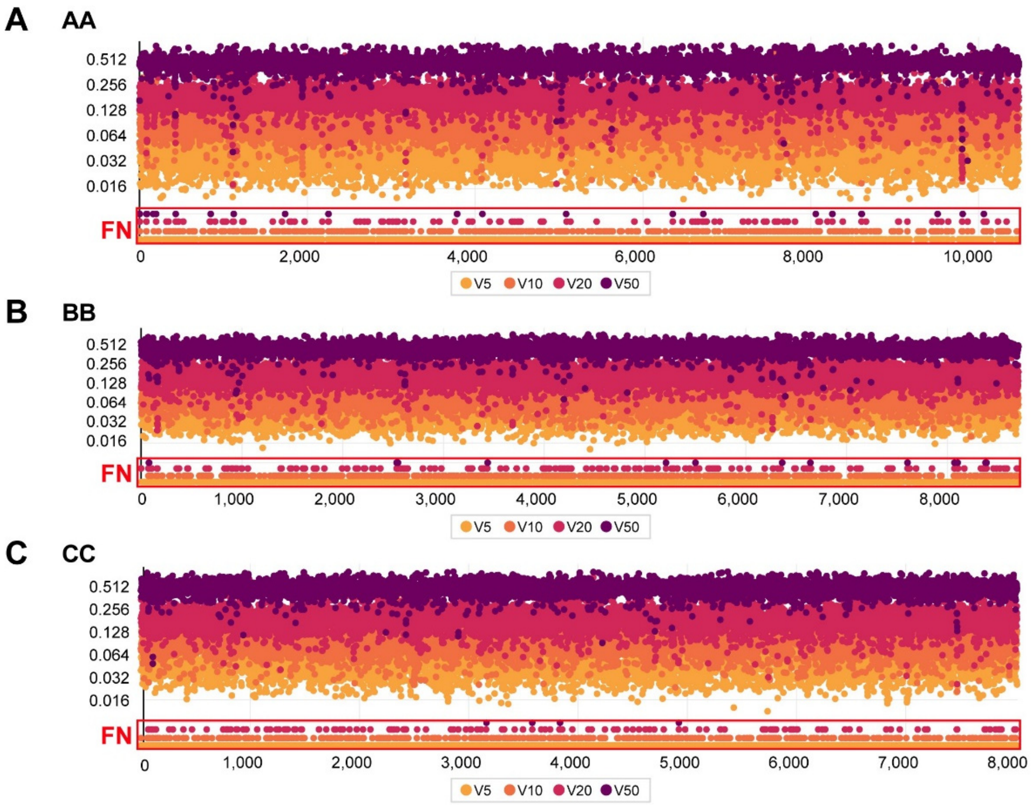

Figure S2. Plot of observed VAF and false negative (FN) errors in N-H variants analyzed by the DRAGEN system in whole exome sequencing (WES) results from three companies. A. Observed VAF and false negative (FN) errors in DRAGEN-analyzed N-H pairs from company AA. B. Observed VAF and FN errors in DRAGEN-analyzed N-H pairs from company BB. C. Observed VAF and FN errors in DRAGEN-analyzed N-H pairs from company CC. The X-axis shows chromosome numbers, while the Y-axis illustrates the observed VAFs in relation to the expected VAFs (V0 to V50).

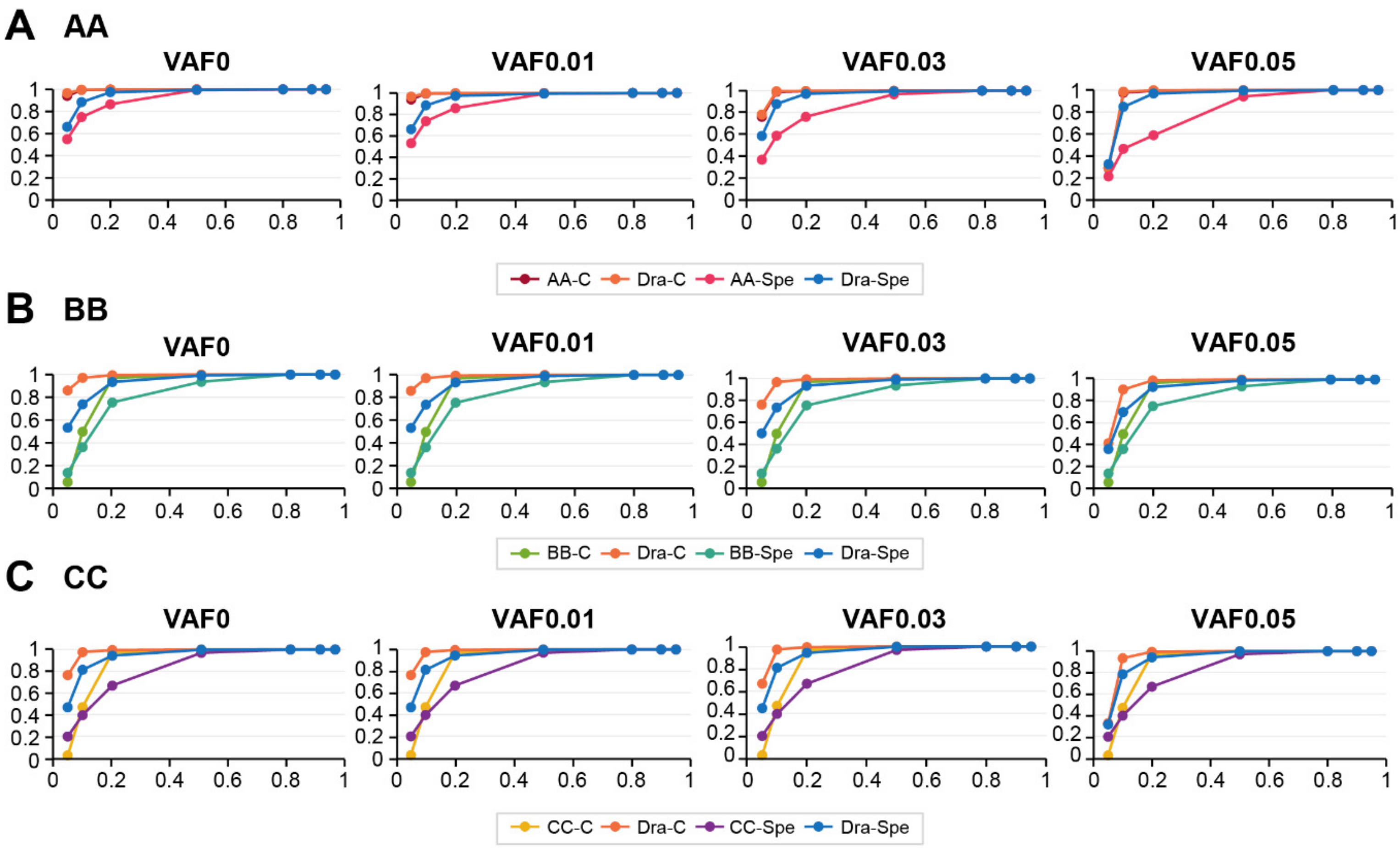

Figure S3. Detection rates of variants across various categories based on different VAF cutoffs. A. Detection rate of variants in AA results according to various VAF cutoffs. B. Detection rate of variants in BB results according to various VAF cutoffs. C. Detection rate of variants in CC results according to various VAF cutoffs. The X-axis represents expected variant allele frequencies (eVAF), while the Y-axis indicates the detection rate of variants. VAF cutoffs ranging from 0 to 0.05 are labeled as VAF0 to VAF0.05. The specific categories Dra-C, AA-C, BB-C, CC-C, Dra-Spe, AA-Spe, BB-Spe, and CC-Spe are described in the Materials and Methods section.

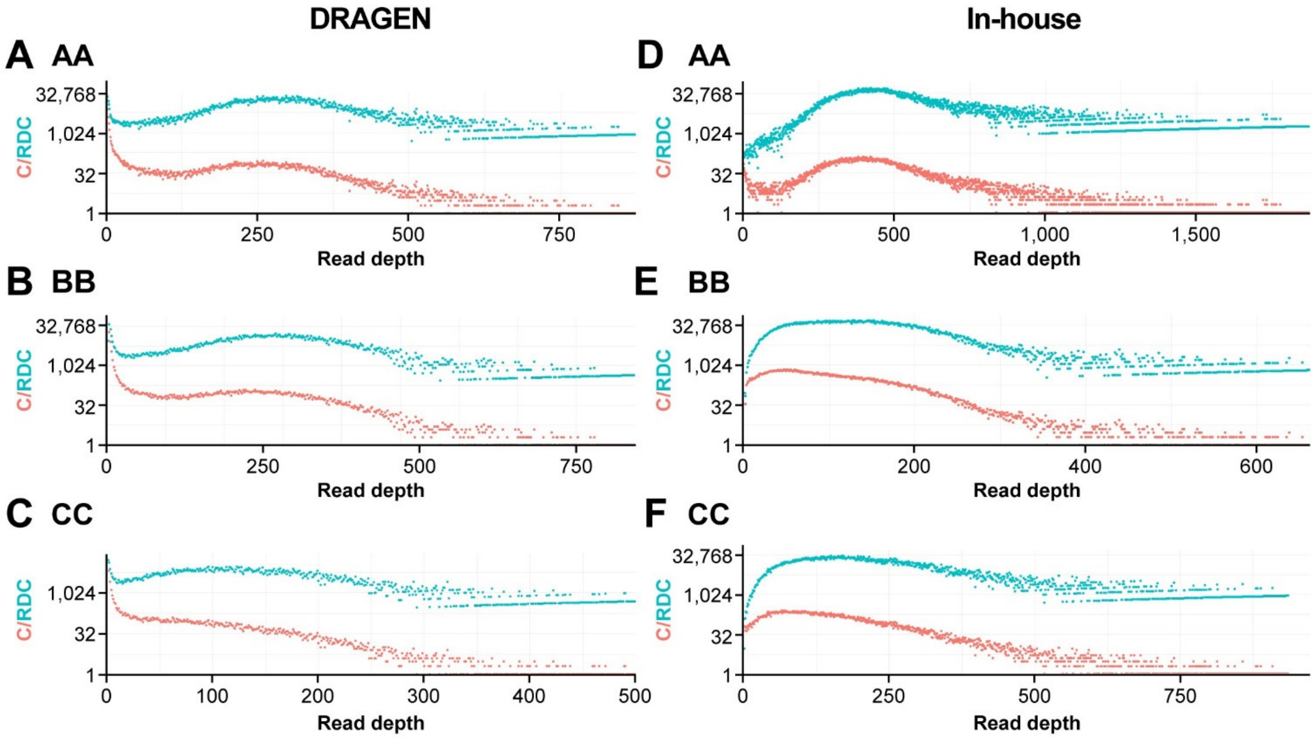

Figure S4. Read depth distributions of variants with the same read depths in DNA2. A. Read depth distributions for DRAGEN-analyzed variants from company AA. B. Read depth distributions for DRAGEN-analyzed variants from company BB. C. Read depth distributions for DRAGEN-analyzed variants from company CC. D. Read depth distributions for in-house analyzed variants from company AA. E. Read depth distributions for in-house analyzed variants from company BB. F. Read depth distributions for in-house analyzed variants from company CC. The X-axis represents the read depth at variants with the same read depth, while the Y-axis displays the count of variants with the same read depths (C, in red), alongside the total read depth count (RDC, in blue) which is calculated by multiplying the count of variants by their corresponding read depth.

Table S1. Total reads, the percentage of bases over Q30, mean depth, reads mapped and paired, reads duplicated, duplication rate, and average read length for in-house WES results from companies AA, BB, and CC.

Company	Sample	Total reads	Q30ratio	Mean depth	Reads mapped and paired*	Reads duplicated*	Duplicated rate*	Average read length (bp)*
AA	DNA1	208,393,314	95.8	397.7841	146,877,873	11,895,258	0.080987406	151
	DNA2	252,502,818	96	468.7917	179,581,115	18,614,804	0.103656802	151
	CH5	237,304,716	94.6	421.9768	161,408,303	15,670,732	0.097087521	151
	CH10	216,906,362	94.7	394.3775	148,463,481	12,886,932	0.086802033	151
	CH20	236,740,964	94.7	432.1612	161,989,033	13,694,406	0.084539093	151
	CH50	210,669,880	95.5	386.3187	145,070,351	11,020,549	0.075966929	151
	CH80	265,869,840	94.6	465.0712	181,334,808	18,185,584	0.100287331	151
	CH90	272,491,716	95.1	489.9764	189,003,329	18,645,212	0.098650178	151
	CH95	194,183,590	95.3	368.726	138,608,385	12,510,872	0.090260571	151
BB	DNA1	141,314,174	93.4	135.5983	89,063,216	0	0	101
	DNA2	142,159,180	93.5	150.9342	95,031,926	0	0	101
	CH5	136,289,952	93.8	125.5837	79,966,555	0	0	101
	CH10	123,317,052	93.0	109.6914	69,778,678	0	0	101
	CH20	123,814,868	93.4	129.9915	83,172,002	0	0	101
	CH50	140,940,508	93.2	132.2735	83,445,056	0	0	101
	CH80	122,850,954	93.9	124.653	79,185,263	0	0	101
	CH90	122,049,230	93.5	104.4226	68,186,721	0	0	101
	CH95	136,071,842	93.4	138.8914	90,198,455	0	0	101
CC	DNA1	106,108,800	94.9	176.6597	73,942,950	0	0	151
	DNA2	115,630,982	94.7	187.0459	79,450,058	0	0	151
	CH5	139,130,706	94.8	188.4894	84,595,264	0	0	151
	CH10	120,617,326	94.9	200.2609	84,329,840	0	0	151
	CH20	131,455,380	94.6	199.4797	84,421,460	0	0	151
	CH50	115,725,440	94.6	188.7195	82,190,920	0	0	151
	CH80	138,146,370	93.8	193.6099	89,939,524	0	0	151
	CH90	156,463,192	95.2	272.0819	116,075,564	0	0	151
	CH95	110,872,694	95.2	162.1212	71,581,392	0	0	151

* In-house information was not been provided and was deduced from the in-house BAM files supplied by the companies.

Table S2. The number of false positive errors in R-V and V-V pairs.

	Companies	Errors	CH5	CH10	CH20	CH50	CH80	CH90	CH95
In-house	AA	R-V	21	22	18	22	15	24	15
		V-V	26	28	33	20	38	34	26
	BB	R-V	4	5	3	4	4	8	4
		V-V	13	11	12	12	11	13	7
	CC	R-V	2	1	1	3	1	1	0
		V-V	1	0	1	2	0	0	0
DRAGEN	AA	R-V	2	2	5	3	1	1	3
		V-V	7	7	13	7	4	7	5
	BB	R-V	3	3	1	1	0	0	0
		V-V	3	2	3	2	1	3	0
	CC	R-V	0	0	0	0	0	0	0
		V-V	2	1	4	2	1	1	1
